# Are urine flow-volume nomograms developed on Caucasian men optimally applicable for Indian men? Need for appraisal of flow-volume relations in local population

**DOI:** 10.4103/0970-1591.70562

**Published:** 2010

**Authors:** Mayank M. Agarwal, Sunirmal Choudhury, Arup K. Mandal, Ravimohan Mavuduru, Shrawan K. Singh

**Affiliations:** Department of Urology, Postgraduate Institute of Medical Education and Research, Chandigarh, India

**Keywords:** Asian, caucasian, healthy male, uroflowmetry

## Abstract

**Introduction:**

Flow-volume nomograms and volume-corrected flow-rates (cQ) are tools to correct uroflow rates (Q) with varied voided volumes (VV) of urine. We investigated the applicability of the available nomograms in our local population.

**Materials and Methods:**

Raw data of our previous study on variation in Q with voiding position (standing, sitting, and squatting) in healthy adult men was reanalyzed. Additionally, the departmental urodynamic database of the last four years was searched for uroflow data of men with voiding symptoms (International Prostatic Symptom Score (IPSS) > 7 and global quality of life score >2). These results were projected on the Liverpool and Siroky nomograms for men. The Q-VV relations were statistically analyzed using curve-estimation regression method to examine the current definition of corrected maximum flow rate (Qmax).

**Results:**

We found a cubic relation between Q and VV; based on this we developed novel equation for cQ [cQ=Q/(VV)^1/3^] and novel confidence-limit flow-volume nomograms. The imaginary 16^th^ percentile line of Liverpool nomogram, -1 standard-deviation line of Siroky nomogram and lower 68% confidence-limit line of our nomogram had sensitivity of 96.2%, 100% and 89.3%, and specificity of 75.3% 69.3% and 86.0%, respectively for Qmax-VV relations. Corresponding values for average flow rate (Qave)-volume relations were 96.2%, 100% and 94.6%, and 75.2%, 50.4% and 86.0%, respectively. The area under curve of the receiver operating characteristics (ROC) curve for cQmax and cQave was 0.954 and 0.965, respectively, suggesting significantly higher discriminatory power than chance (*P* = 0.0001).

**Conclusion:**

Flow-volume nomograms developed on Caucasian population may not be optimally applicable to the Indian population. We introduce flow-volume nomograms and cQ, which have high sensitivity and specificity.

## INTRODUCTION

Uroflowmetry is the most commonly utilized urodynamic investigation. It is a non-invasive tool to screen patients with voiding dysfunction. Flow rates are affected by many factors, including voided volume, age, psychological inhibition, and abdominal straining, etc.[1] This leads to considerable overlap between normal and abnormal population. Flow–volume nomograms[2–5] and volume-corrected flow rates[6] have been developed to evaluate flow rates over varied voided volumes, ages and both sexes.

Racial and ethnic differences have been reported to account for difference in various normal as well as disease parameters.[7–9] The issue is less well studied in Urological science; however, there are some reports on differences in prostate size,[10] incidence of benign prostatic hyperplasia (BPH),[11] behavior of prostate cancer,[12] uroflow rates (men and women)[2–10] and urinary incontinence.[13] Since there are clear demographic differences in the Caucasian and Asian population, and based on our personal observation in clinical practice as well as published experience in women,[2] we suspected applicability of available flow-volume nomograms developed for Caucasian men on ours. The study was conducted to examine the same in our male population.

## MATERIALS AND METHODS

### 

The study protocol was approved by the institution ethics committee. We used raw database and results of our previously published study.[14] Details of participants’ demographic and uroflowmetric data have been reported in our earlier publication.[14] Briefly, healthy male volunteers with mean age 26.6 ± 6.9 years and body mass index 21.9 ± 3.4 kg/m^2^ without any lower urinary tract symptoms (LUTS) as assessed using International Prostatic Symptom Score (IPSS) were enrolled. Each participant was asked to report with comfortably full bladder and to void into a digital gravimetric uroflowmeter (Digital Urodynamic Machine, Solar Silver, MMS International, Enschede, The Netherlands) twice in three different voiding positions, i.e. standing, sitting and squatting (all voids at separate times without randomization to any sequence). Only representative continuous voids were included for data analysis. The studies were performed and interpreted in compliance with the guidelines of the Standardization Sub-committee of the International Continence Society (ICS).[15] Flow-volume nomograms were constructed on this data using Statistical Package for Social Sciences (SPSS, Version 16, USA).

For testing the discriminating power of flow-volume nomograms, data of adult men with bothersome voiding type LUTS (poor stream, straining at micturition, sensation of incomplete evacuation, intermittency and hesitancy) seen at our clinics was included. It was retrieved from the institution’s urodynamic database; using the ‘query’ module of the urodynamic software we searched for “uroflowmetry” of “men” in patient categories “LUTS”, “BPH” over the last three years. The clinical history of each of these patients was reviewed from the “investigation memo” of the investigation. Data of only those patients having a written record of IPSS score more than 7 and global quality of life score more than 2 and minimum voided volume of 150 ml was included.

The following urine flow-volume nomograms were included for comparison with our results:

1. Liverpool Qmax-volume and Qave-volume nomogram for men younger than 55 years[3]
2. Siroky Qmax-volume and Qave-volume nomogram (ref) for men under age 50 years.[4]

For uniform comparison of the status of our plotted data between the above nomograms, imaginary lines were drawn on the Liverpool nomogram at 16^th^ and 2.5^th^ percentile (roughly equivalent to −1 SD and − 2 SD of the Siroky nomogram). Additionally, the von Garrelt definition[6] of corrected Q (cQ = Q/√VV) was used for testing on our population.

#### Statistical analysis

All the data was fed into Microsoft Excel worksheet and analyzed using statistical package for social sciences for Windows (SPSS, Version 16, USA). Continuous variables were presented in mean ± standard deviation (median, range) and categorical variables in percentages. Normality of distribution of data was tested using 1-sample Kolmogorov-Smirnoff test and was found to be non-normal. Therefore, flow-volume correlations were tested using Spearman’s rho for ranks. For purpose of flow-volume nomograms several transformations of data were assessed and the goodness-of-fit tested to determine whether a linear, quadratic, cubic, or logarithmic function best described the relation between the maximum flow rate (Qmax) and average flow rate (Qave) and voided volume (VV). For presentation, the nomograms have been expressed in the form of median, and 68% and 95% confidence limits. The diagnostic utility of the nomogram was tested by plotting data of normal controls and the symptomatic patients on the nomograms. The utility of cQmax/ave was estimated by calculating area under curve (AUC) in receiver operating characteristics (ROC) curve.

## RESULTS

### 

#### Data consideration

Data of 61 participants of the above mentioned study[14] was included for this study. Their mean age was 26.9 ± 6.9 years (25; 18-45) and body mass index 21.9 ± 3.4 kg/m^2^ (21.6; 15.8 - 32.6). All men were accustomed to voiding in standing position; 45 preferred standing position, 15 preferred squatting and only one sitting. Mean Qmax was 23.8 ml/sec in standing, 24.4 in squatting and 19.8 in sitting position (mean VV 350, 359 and 363 ml, respectively). The corresponding values for Qave were 13.9, 13.8 and 11.2, respectively. There was no significant difference between voiding in standing and squatting position (*P*=0.55); whereas, the difference was significant between either of these positions with sitting position (*P*=0.0001). We, therefore, included the uroflow data recorded in the former two positions only (total 244 uroflow results).

Data of 57 patients with mean age 54.5±21.3years (median 60.0; range 12-92) with bothersome voiding LUTS was included. Their Qmax, Qave and VV were 10.5±3.9 ml/sec (10; 4-19), 5.3±2.3 ml/sec (5; 2-12) and 305.3±150.8 ml (265; 159-973).

#### Volume-corrected flow rates

There was significant correlation between flow rates and VV –

1. Qmax – VV: *P* = 0.0001; Spearman’s rho = 0.263
2. Qave – VV: *P*=0.0001; Spearman’s rho = 0.333
3. Qmax and Qave had a cubic relation with VV (Q α VV^3^) as follows:
  Qmax = 0.712 + (0.154 X VV) + (0.0001 X VV^2^) + [(1.949E-7) X VV^3^]
  Qave = 2.199 + (0.067 X VV) + (0.0001 X VV^2^) + [(5.690E-8) X VV^3^]

We, therefore, developed the following definitions for volume-corrected flow rates:

1. C_Qmax_= Qmax/VV^1/3^
2. C_Qave_= Qave/VV^1/3^

We found the quadratic corrected flow rates to significantly correlate with VV (*P*=0.0001; spearman’s rho for cQmax-VV = -0.34, cQave = -0.27). The correlations between our novel cQ and VV were statistically significant for cQmax with lower degree of correlation (*P*=0.02, cc = -0.14) and insignificant for Qave (*P*=0.33, cc = -0.06) suggesting their superior clinical significance in our population compared to quadratic one.[6]

### Flow-volume nomograms

Flow-volume nomograms (eponymed as PGIMER nomogram, based on our institution’s acronym) were constructed for Qmax and Qave separately. Third order polynomial trendlines for mean, 68% confidence limits (CL) and 95% CL were projected on the scatter plots with flow rates on Y axis and voided volume on X axis to construct the nomograms (since Q-VV relations were cubic in our population).

**Figure 1:**
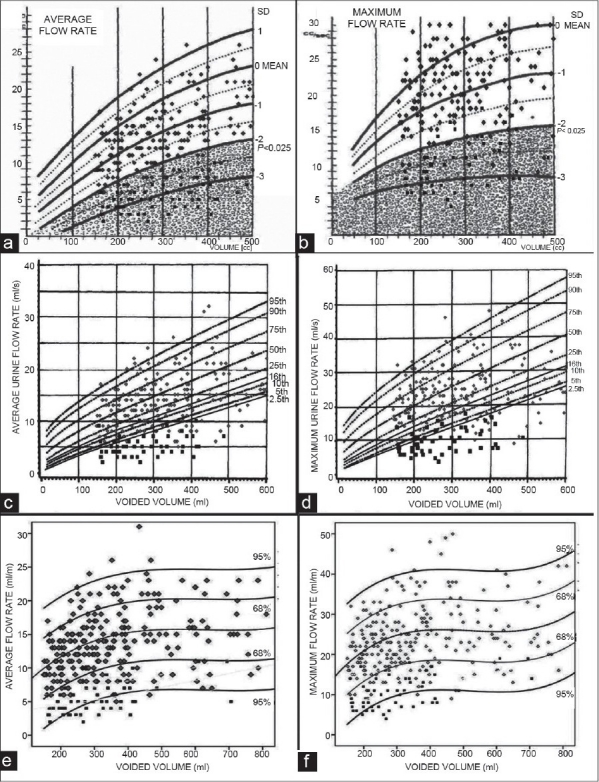
Plotting of our normative (
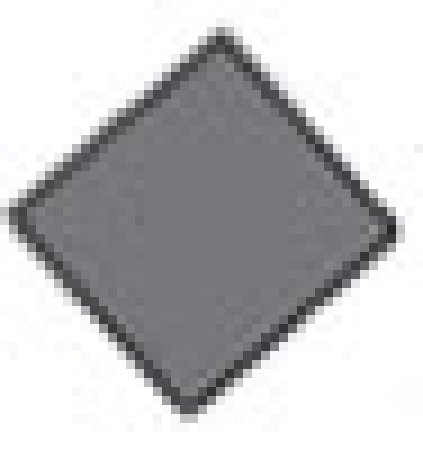
) and abnormal (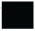) results on Siroky (a, b), Liverpool (c, d) and flow-volume nomograms developed by us (e, f; PGIMER nomograms)

**Figure 2:**
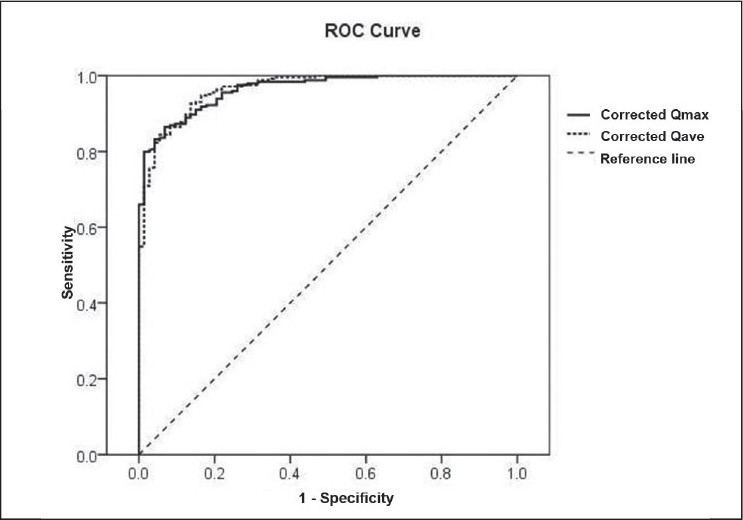
Receiver-operating-characteristics curves drawn for cQmax and cQave as per our novel defi nition i.e. cQ = flow-rate/(voided volume)1/3. The AUC was 0.955 & 0.965, respectively (*P*=0.0001) pointing towards high sensitivity and specifi city of cQ in evaluating flow rates.

Our normal and abnormal results were plotted on Liverpool,[3] Siroky[4] and PGIMER nomograms. The imaginary line of the 16^th^ percentile (Liverpool), −1SD (Siroky) and lower 68% CL (PGIMER) represented statistically equivalent lines, so did the imaginary line of the 2.5^th^ percentile, −2SD and lower 95% CL, respectively. Plotting of flow-volume data of healthy participants and symptomatic patients on all the three nomograms [Figure 1] revealed higher sensitivity but lower specificity of Liverpool and Siroky nomograms compared to the PGIMER nomogram [Table 1]. The ROC curve [Figure 2] drawn for corrected Qmax and corrected Qave showed an AUC of 0.954 and 0.965, respectively (*P*=0.0001).

**Table 1:** sensitivity and specificity of various flow-volume nomograms with respect to two levels of cutoffs

	−1SD (S), 16th percentile (L) and lower 68% CL (P) line		−2SD (S), 2.5th percentile (L) and lower 95% CL (P) line	
	Sensitivity	Specificity	Sensitivity	Specificity
	Mean % (lower-upper limit; 95% confidence interval)			
For qmax-voided volume relations				
Siroky nomogram (S)	100 (91.7-100)	69.3 (62.3-75.4)	85.2 (72.3-92.9)	93.1 (88.4-96.0)
Liverpool nomogram (L)	96.2 (85.9-99.3)	75.6 (69.3-81.1)	79.2 (65.5-88.7)	90.4 (85.4-93.8)
PGIMER nomogram (P)	89.3 (77.4-95.6)	86.0 (80.9-89.9)	25.9 (15.3-39.9)	99.2 (96.7-99.9)
For qave-voided volume relations				
Siroky nomogram (S)	100 (91.7-100)	50.4 (43.4-57.5)	96.2 (86.2-99.2)	85.1 (79.3-89.6)
Liverpool nomogram (L)	96.2 (85.9-99.3)	75.2 (68.8-80.7)	88.6 (76.2-95.3)	91.3 (86.5-94.5)
PGIMER nomogram (P)	94.6 (84.2-98.6)	86.0 (80.9-89.9)	28.6 (17.7-42.4)	99.2 (96.7-99.9)

## DISCUSSION

‘Racial and ethnic differences’ have been reported to account for difference in various normal as well as disease processes.[7–9] Personal demographics (height, weight, etc.), food habits, socioeconomic status as well as environment have been reported to account for the ‘racial and ethnic differences’. The issue is less well studied in urology; nevertheless, there are some small reports suggesting differences in prostate size,[10] incidence of BPH,[11] behavior of prostate cancer,[12] uroflow rates (men and women)[2–10] and urinary incontinence[13] across races. Masumori **et al**.[10] compared prostate volume and peak urine flow rates of 286 Japanese men (mean age 61.3 years) living in Shimamaki-mura village to 471 of those living in Olmsted county in the USA (mean age 55.6 years). In all age groups, mean prostate size of the latter group was higher than the former by 61-93%. Peak flow rates were higher in Japanese men younger than 60 years, compared to age-matched Americans; the reverse was true in older men.

**Figure 3:**
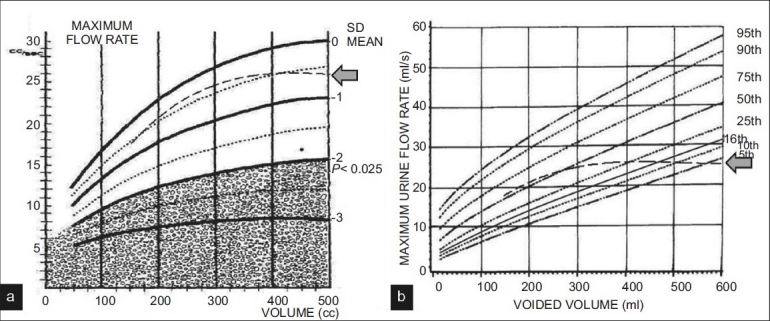
status of 3rd order polynomial trendline of our data as plotted on (a) Siroky nomogram & (b) Liverpool nomogram for Qmax. Location of the trendline is marked by block arrow (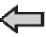)

In this study, we found that uroflow rates of young adult men in our country were lower than the corresponding values of Caucasian men. Therefore, a significant number of normal flow-volume relations of our men fared poorly on flow-volume nomograms developed on American (Siroky nomogram) or British men (Liverpool nomogram) [Figures 2 and 3]. We also found that the definition of volume-corrected Qmax (cQmax), first described by von Garrelts[6] based on quadratic Q-VV relations, did not hold true for our population since cQ computed based on that definition had positive correlation with VV; it should have been independent / poorly correlating with the latter. Therefore, based on cubic flow-volume relations of our population, we developed a new definition which fared better as evaluated by AUC in ROC curve [Figure 2]. We also introduced the term corrected cQave in men for the first time in the literature. This term was earlier introduced for women by our group.[2] Notably, in that study, Q-VV relations were quadratic and therefore as expected the Von Garrelt definition of cQ was found to be valid. Qave is an important parameter while interpreting uroflow data, and is an indicator of overall flow quality, which may at times be overestimated if one looks only at Qmax, and not consider flow curve and Qave. Similar to Qmax, Qave is dependent on VV. Therefore, considering Qave to be important and its dependency on VV, we introduced the concept of volume-corrected Qave (cQave) for women as well as men. Further studies are required to further validate this concept.

Since urine flow rates are affected by many factors, including VV, age, sex, voiding position, psychological inhibition and abdominal straining, etc,[1–14] there is considerable overlap between normal and abnormal population. Moreover, there are a few reports to suggest a demographic difference in uroflow rates. Masumori *et al*.[10] reported different age-related trends of uroflow rate in Japanese (n=286) and American men (n=471). Japanese men younger than 55years showed a trend of higher predicted Qmax, which reversed in older men. Estimated decrease in Qmax with age was twice as high as in the former group. Barapatre *et al*.[2] compared sitting uroflow rates in Indian women (n=308) with historical controls (Liverpool data; n=249) and found significantly lower flow rates in age-matched Indian women.

The most obvious demographic differences between the Asian and Caucasian population are those in height and weight. According to the third American national health and nutrition examination survey (NHANES III), the average height of an American man was 177 cm and weight 81 kg. Masumouri *et al*.[10] reported similar results for American men in their study, compared to 163 cm and 63 kg, respectively for Japanese men. Average height and weight of men in our study was 166 cm and 61 kg, respectively which correspond to our national average. This may partly explain the difference in uroflow parameters among various populations. We found a statistically significant correlation of height and weight with uroflow parameters; however, the correlation of body mass index (BMI) was insignificant. Masumouri *et al*.[10] reported that variation in height, weight, volume and age accounted for 41% of variability in uroflow rates; BMI was not reported and the contribution of the former two was not separately mentioned. We earlier reported (Barapatre *et al*.)[2] a significant effect of height, weight and BMI on uroflow rates in young women. The effect of body indices are likely to be more pronounced in women than men due to difference between voiding physiology; a significant proportion of normal women void predominantly by urethral relaxation with little contribution from the detrusor, a phenomenon unknown in normal men. Overall, height and weight, mostly speculatively, may partly account for the differences in uroflow rates among various races.

The difference in the flow-volume relations at different volumes may also explain the difference between nomograms. The cubic trendline of our data corresponded to the 50^th^ percentile line of the Liverpool nomogram and (nearly) mean line of the Siroky nomogram at volumes approximately 200-250 ml; however, it dipped progressively at higher volumes [Figure 3]. Our results indicate relatively constant flow rates between VV 400-600 ml (trendlines nearly horizontal); whereas, the curves on the Western nomograms[3,4] are ascending at all available volumes, more so in the Liverpool nomogram. The efficiency of the detrusor tends to decrease beyond volumes more than 400 ml adversely affecting flow rates,[15] therefore our results corroborate with the existing literature on bladder physiology. Although the reason of continuous escalation on the Western nomograms is poorly understood, the strength of the database used may have a bearing. The mean VV in the Liverpool group was 190 ml suggesting that only few observations would be >400 ml; therefore, the resulting nomogram at those volumes is likely to be influenced by the trend of preceding volumes.

Higher sensitivity of Western nomograms[3,4] in detecting abnormally low flow rates in our population is understandable since their normality standards are set at higher levels, as evidenced by our normality trendline falling below the mean of these nomograms. Notably, this higher sensitivity resulted in lower specificity [Table 1].

Since, uroflowmetry is the pivotal initial investigation in the management of voiding dysfunction, its accurate interpretation becomes even more crucial. As is evident from the above discussion, there are at least apparent differences in flow rates between the Asian and Caucasian population. Therefore, it may not be fully appropriate to interpret our results on these nomograms. This calls for studying the flow-volume characteristics of the race in question before applying any available nomograms or corrected flow rates.

One important limitation of our study was its retrospective nature and cumulating data acquired by repeat voids in two different positions on a relatively small number of patients. Nevertheless, we did prove similarity between voiding characteristics of the two voiding positions (standing, squatting) prior to inclusion and also excluded data of sitting position which was dissimilar. Moreover, the higher specificity of our nomogram and large AUC in the ROC for corrected Q points towards at least preliminary validity and clinical utility of both the concepts (namely the PGIMER nomograms and cubic corrected Q). However, a study with larger number of participants will be required for further validation. The strength of our study was to perform simplistic validation of the nomograms by plotting abnormal results from symptomatic patients, increasing their applicability. Although all the symptomatic patients had not undergone multichannel cystometry for confirming presence of bladder outlet obstruction / detrusor underactivity, the data remains clinically relevant since uroflowmetry itself is a non-invasive investigation.

## CONCLUSION

Currently flow-volume nomograms developed on the Caucasian population are not optimally applicable to the Asian population. We developed preliminary PGIMER nomograms on our population and found them to be superior in discriminating normal from abnormal flow-volume relations. We also presented a modified definition of volume-corrected flow rates.
